# Empiric antibiotic regimen for febrile neutropenia in cancer patients: a tertiary oncology center experience

**DOI:** 10.3389/fonc.2026.1876292

**Published:** 2026-07-29

**Authors:** Hasan Arafat, Marah Sabateen, Dana Sadaqa, Hammam Rjoup, Ayman Shoeibat, Musa Hindiyeh, Ali Sabateen

**Affiliations:** 1 Department of Internal Medicine, Augusta Victoria Hospital, East Jerusalem, Palestine; 2 Department of Obstetrics and Gynecology, Holy Family Hospital, Bethlehem, Palestine; 3 Infectious Disease Unit, Augusta Victoria Hospital, East Jerusalem, Palestine; 4 Department of Laboratory Medicine, Augusta Victoria Hospital, East Jerusalem, Palestine

**Keywords:** antibiotic stewardship, fever, immunocompromised, malignancy, neutropenic fever

## Abstract

**Introduction:**

Neutropenia is a major obstacle in the care of cancer patients. It is common in patients receiving chemotherapy, associated with significant morbidity and mortality, it occurs in 5-30% among patients with solid tumors and exceeds 80% of those undergoing chemotherapy for hematologic malignancies.

**Objectives:**

This study primary endpoint was to assess the clinical outcome of the different antibiotic regimens. The secondary endpoint was to assess the microbiological appropriateness of antibiotic coverage of our carbapenem-sparing empiric regimen for neutropenic fever in patients admitted to the hemato-oncology departments to Augusta Victoria Hospital.

**Methods:**

Patients data were extracted from the hospital health information system for all entries pertaining to neutropenic fever from 1 January 2022 to 30 November 2025. Statistical comparison between empiric antibiotic regimens was not performed because treatment escalation followed a predefined sequential protocol, resulting in non-independent groups. Assessment of antimicrobial appropriateness was completed using IBM SPSS Statistics via Fisher’s Exact Test. Patient data analyzed, included antibiotic classes used, bacterial culture results, antimicrobial sensitivity testing, cause of fever and outcome of antimicrobial treatment. Exclusions criteria included prophylaxis prescriptions for neutropenic fever and pediatric patients.

**Results:**

Overall, 208 patients with febrile neutropenia were evaluated between January 1st, 2022, to November, 2025^th^ 2025. Hematology patients represented a major proportion compared to oncology (56.70% and 43.30%, respectively). Our institutional protocol, represented by ceftazidime and amikacin (the last is replaced with ciprofloxacin for patients with renal failure) was the most prescribed regimen (78.1%), followed by piperacillin-tazobactam ± vancomycin (16.4%) and finally meropenem (5.5%). Our institutional regimen was theoretically appropriate in 44.4% of cases, compared to piperacillin-tazobactam ± vancomycin (55.6%) and meropenem (88.9%).

**Conclusion:**

The institutional stepwise empiric antibiotic protocol demonstrated high overall clinical response rates in patients with febrile neutropenia, with most patients achieving improvement without requiring escalation beyond first-line therapy. Simulated microbiological assessment suggested increasing pathogen coverage with broader-spectrum regimens, although this should be interpreted cautiously given the limited number of positive culture isolates. These findings support the effectiveness of the current protocol while emphasizing the importance of balancing microbiological coverage with antimicrobial stewardship principles.

## Introduction

Febrile neutropenia (FN) following chemotherapy is common. Due to its associated with a high mortality rate, designation of an empiric, broad spectrum antibiotic regimen, with *Pseudomonas aeruginosa* coverage is considered a cornerstone due to the high mortality and morbidity associated with that organism (1). Evidence accumulated suggests that adequate coverage can only be better achieved by using combination antibiotics. Therefore, current protocols suggest using either a β-lactams with an aminoglycoside or two classes of β-lactams together. On the other hand, fear of toxicity led to the emergence of alternative protocols that suggest using a single, broad spectrum β-lactams (2). Therefore, no single regimen can be unequivocally recommended for FN, and the choice of antibiotic therapy remains mainly based on local antibiotic protocols (1).

The Infectious Disease Society of America (IDSA) and the American Society of Clinical Oncology (ASCO) recommends treating patients with FN according to their risk. High-risk patients require hospitalization for IV antibiotics. Monotherapy using a broad-spectrum, anti-pseudomonal β-lactam is recommended. Agents recommended included cefipime, a carbapenem (meropenem or imipenem) or piperacillin/tazobactam. Other agents (such as aminoglycosides, fluoroquinolones and vancomycin) can be added to the regimen for management of complications or if antimicrobial resistance is proven (3). For low-risk patients, outpatient oral antibiotics are recommended, the recommended regimen includes a fluoroquinolone (ciprofloxacin/levofloxacin) plus amoxicillin/clavulanate, clindamycin can be substituted for those who are penicillin-allergic (4). The European Society of Medical Oncology (ESMO), on the other hand, shared the same view regarding outpatient therapy, but recommended using combination therapy for high-risk patients, consisting of a β-lactam (ceftazidime) plus an aminoglycoside, as their synergistic effect proved beneficial, especially in patients with prolonged neutropenia or bacteremia (5) Nevertheless, the use of aminoglycosides in FN remains hindered by the risk of nephrotoxicity. A systematic review by Paul et al. found the risk of aminoglycoside-induced nephrotoxicity was limited to 1%, however, risk of nephrotoxicity was increased with prolonged use of aminoglycosides, in addition to multiple daily dosing in comparison with single dose per day (6).

Novel β-lactams with broader coverage have been studied in the context of rising prevalence of multi-drug-resistant organisms (MDRO), however, empirical use of these agents is not recommended. Appropriate therapy choices should be determined by the patient’s presentation, previous culture data, and the use of institutional antibiograms (7).

Current guidelines recommend assessing the risk for FN prior to each chemotherapy cycle (8). When it does occur, IDSA/ASCO guidelines recommend risk stratification via the Multinational Association for Supportive Care in Cancer (MASCC) scoring system, a high risk patient (MASCC < 21) should be initially admitted to hospital for IV antibiotics. Low risk patients (MASCC ≥ 21) can be selectively treated as outpatient (3). Other scoring systems do exist, but a comprehensive comparison between existing systems is beyond the scope of this manuscript.

Introduced 11 years ago, our institute’s first-line recommended empiric antimicrobial regimen for FN (ceftazidime and amikacin) has remained unchanged despite the rise of antibiotic resistance (9). In this study, we evaluate the adequacy of coverage provided by our protocol against bacterial isolates from patients admitted with FN and to compare it to other guideline-recommended protocols.

## Methods

### Patient population

This is a retrospective cohort study that was conducted at Augusta Victoria Hospital, a tertiary hospital specialized in oncology, hematology and dialysis services. Adult patients with cancer who were admitted to the medical oncology, hematology, bone marrow transplantation and intensive care unit at Augusta Victoria Hospital between January 1^st^, 2022, and November, 2025,^th^ 2025 were included as the target population for this study. Each new admission for the same patient was counted as a new case. All consecutive eligible patients meeting inclusion criteria during the study period were included; sample size was determined by available retrospective data.

Missing data were assessed for completeness prior to analysis. Variables with missing values were reported, and analyses were performed using available-case (complete-case) analysis, whereby only observations with available data for the variable of interest were included. No imputation was performed due to the retrospective nature of the study and the limited proportion of missing data. Cultures that grew fungi were not included in positive culture analysis. The number of available observations for each analysis is reported where applicable.

### Antibiotic protocol

At our institution, the empiric antibiotic protocol for the management of febrile neutropenia follows a stepwise escalation approach. First-line therapy consists of combination treatment with a broad-spectrum β-lactam, ceftazidime, in addition to an aminoglycoside, amikacin. In patients with impaired renal function, amikacin may be substituted with ciprofloxacin. In cases of clinical non-response or deterioration, therapy is escalated to a second-line regimen comprising broad-spectrum monotherapy with piperacillin/tazobactam, with the addition of vancomycin in selected patients based on clinical indications such as suspected Gram-positive infection. For patients who fail to respond to second-line therapy or who present with more severe or resistant infections, escalation to a third-line regimen with meropenem, representing the broadest spectrum coverage in our protocol, is undertaken.

### Laboratory testing

Aerobic and anaerobic blood cultures were collected in VersaTREK blood culture bottles and incubated according to the manufacturer instructions (Thermo Fisher Diagnostics, USA). All positive blood cultures obtained for suspicion of infection (with or without fever) from eligible population obtained within the first 48 hours of admission were evaluated for inclusion. Cultures growing common skin bacteria suspected for blood culture contamination were excluded unless the same isolate was obtained from two consecutive blood cultures at two different sites.

Positive blood cultures were included in the analysis if they were obtained from patients who were concomitantly neutropenic. Our definition for FN was the presence of single oral temperature greater than or equal to 38.3 °C or a temperature greater than or equal to 39 °C for at least an hour, with an absolute neutrophilic count (ANC) of less than 500 cells/µL or an ANC of ≤ 1000 cells/μ L that is predicted to drop below 500 cells/μ L within the next 48 hours. [1, 2]. Severe neutropenia is less than 500 per microliter. In profound neutropenia, the ANC is less than 100 cells/microliter (10).

### Outcomes

The primary endpoint was clinical appropriateness of empiric antibiotic therapy, defined as attainment of clinical response characterized by defervescence, overall clinical improvement, and recovery from neutropenia. The secondary endpoint was microbiological appropriateness, defined as the theoretical adequacy of coverage provided by the evaluated antibiotic regimens against pathogens isolated from positive blood cultures. Susceptibility was assessed based on the Clinical and Laboratory Standards Institute (CLSI) standards. We assessed the following antibiotic protocols: (a) The local institutional protocol, ceftazidime and amikacin; (b) a modified institutional protocol for patients with renal impairment, in which ciprofloxacin is substituted for amikacin, also labeled as local institutional protocol; (d) piperacillin/tazobactam, plus vancomycin for eligible patients, such as patients presenting initially with line sepsis, nosocomial soft tissue infection and septic shock; (e) meropenem.

### Ethical approval

The Augusta Victoria Hospital Institutional Review Board (IRB) approved the study, individual informed consent was waived. Approval to access data was provided by the administrative board of Augusta Victoria Hospital.

### Statistical analysis

Statistical comparison of clinical outcomes (the primary endpoint) between antibiotic regimens was not performed, as the treatment protocol followed a predefined sequential escalation strategy rather than independent treatment allocation. Patients who did not respond to first-line therapy were escalated to subsequent regimens; therefore, the treatment groups were inherently dependent and did not meet the assumptions required for comparative inferential analyses. Antimicrobial appropriateness rates were compared using Fisher’s Exact Test. Hypothesis test results were considered statistically significant when two-sided *P*-values were <0.05.

## Results

### Demographic and clinical characteristics of the study population

The study period included 208 patients who fulfilled the above-mentioned definition of FN. 40.4% of patients were females, most of the studied patients belonged to elderly age group (39.9% above 60 years of old). Of these, only 18 had positive blood cultures, with 17 isolating a single bacterial strain.

Among the patients studied in our study, 27.9% of patients had acute myeloid leukemia (AML), followed by multiple myeloma (10.6%) then Hodgkin lymphoma (10.6%), making hematological malignancies the majority of patients affected and included in our study. Baseline demographic characteristics are outlined in **Table 1**.

**Table 1:** Baseline demographics and disease characteristics.

Patient characteristic	N = 208
Female, No. (%)	84 (40.4%)
Age Group Years, No (%)	
19-39	59 (28.4%)
40-59	66 (31.7%)
60-74	72 (34.6%)
≥75 years	11 (5.3)
Treatment Line Used, No (%)	
First Line (Ceftazidime + Amikacin/Ciprofloxacin	155 (74.52%)
Second Line (Piperacillin-Tazobactam ± Vancomycin)	35 (16.83%)
Third Line (Meropenem)	15 (7.21%)
Focus of Infection, No (%)	
Mucositis	51 (38.1%)
Pneumonia	20 (14.9%)
Skin and Soft Tissue Infection	15 (11.2%)
Bacteremia of Unknown Source	14 (10.5%)
Intraabdominal Infection	14 (10.5%)
Herpes Simplex Virus	7 (5.2%)
Invasive Aspergillosis	4 (3.0%)
Urinary Tract Infection	4 (3.0%)
Dental Infection	3 (2.2%)
Neutropenic Vulvovaginitis	1 (0.7%)
Otitis Media	1 (0.7%)

### Isolates in the blood of neutropenic patients on admission

*Escherichia coli* was the most frequent isolate among studied patients (six isolates, 33.3%), followed by *Klebsiella pneumoniae* (22.2%), *Streptococci* species (18.8%), *Staphylococci* species (11.2%; one *S. aureus* and one coagulase-negative *Staphylococcus*), *Burkholderia cepacia* (11.1%), *Corynebacterium jeikeium* (5.6%) and *Enterococcus faecium* (5.6%).

There were seven isolates of extended-spectrum beta lactamase (ESBL) *Enterobacterales*, four *Klebsiella pneumoniae* and three *Escherichia coli*.

### Antibiotic protocol appropriateness rates

Using manual chart analysis, we found that our default first line institutional empiric antibiotic protocol (ceftazidime-amikacin/ciprofloxacin) for FN was prescribed in 78.1% of cases, single agent broad spectrum beta-lactam regimen (piperacillin-based), plus/minus vancomycin according to clinical assessment was used in 16.4% of cases. Carbapenem-based regimen was used in 5.5% of cases.

### What is the appropriateness rate of the used protocol

The present analysis evaluated whether the empiric antibiotic regimens used were effective in covering positive culture results (N = 18). Overall, high coverage rates were observed across the evaluated regimens. When simulating the potential coverage for all isolated pathogens, Regimen 1 (Ceftazidime combined with Amikacin, Ciprofloxacin for kidney injury) demonstrated the lowest coverage rate, 8 out of 18 cases (44.4%, *p* = 0.005) followed by Regimen 2 (Piperacillin/Tazobactam ± Vancomycin) (10, 55.6%, *p=*0.005) while Regimen 3 (Meropenem) showed the highest coverage rate (16, 88.9%, *p* = 0.150). Despite these findings, microbiological coverage should be balanced against antimicrobial stewardship considerations and not be interpreted as a direct surrogate of clinical effectiveness. These results are demonstrated in **Table 2**.

**Table 2:** Simulated coverage rates of evaluated empiric antibiotic regimens (N = 18).

Regimen	Adequate coverage N (%)	Inadequate coverage N (%)
Regimen 1 (Ceftazidime + Amikacin or Ciprofloxacin)	8 (44.4%)	10 (0.55.6%)
Regimen 2 (Piperacillin/Tazobactam ± Vancomycin)	10 (55.6%)	8 (44.4%)
Regimen 3 (Meropenem)	16 (88.9%)	2 (11.1%)

This table represents the theoretical *in-vitro* susceptibility of the 18 isolated pathogens against each regimen.

When evaluating the actual empiric therapy administered to the 18 culture-positive patients, eight patients (44.4%) had their prescribed empiric antibiotic regimen adequately covering the cultured pathogen, while 10 patients (55.6%) received suboptimal coverage. Due to the small sample size, Fisher’s Exact Test was utilized instead of the Pearson Chi-Square test to assess statistical significance. These results are demonstrated in **Table 3**. These results do not demonstrate actual microbiological coverage, but they aim to show that, although our empiric protocol is narrower in coverage compared to both protocols 2 and 3, it is non-inferior as a first choice and aligns with our carbapenem-sparing approach.

**Table 3:** Association between the administered empiric regimen and actual pathogen coverage.

Administered regimen	Coverage inadequate N (%)	Coverage adequate N (%)	Total patients on regimen N (%)
Ceftazidime-Amikacin/Ciprofloxacin	8 (44.4%)	8 (44.4%)	16 (88.9%)
Piperacillin-Tazobactam± Vancomycin	1 (5.6%)	1 (5.6%)	2 (11.2%)
Total	9 (50.0%)	9 (50.0%)	18 (100.0%)

*Calculated using Fisher’s Exact Test. A *p*-value < 0.05 is considered statistically significant.

Type of Cancer in Febrile Neutropenic Fever.

A statistically significant difference in microbiological coverage was observed across the three antibiotic protocols, with coverage increasing sequentially from protocol 1 to protocol 3. This suggests that later-line regimens provided broader theoretical activity against isolated pathogens than earlier protocols. Given the sequential and non-random allocation of antibiotic regimens, the observed increase in microbiological coverage should be interpreted as reflecting the expected broadening of antimicrobial spectrum across escalation steps rather than evidence of comparative clinical superiority. Fisher’s Exact Test demonstrated a statistically significant association between regimen and theoretical microbiological coverage; however, this finding should be interpreted with considerable caution because only 18 culture-positive cases were available and regimen allocation was highly imbalanced.

## Discussion

This study evaluated cases of neutropenic fever admitted to the hemato-oncology departments of a single tertiary referral center. Our primary endpoint was to assess the appropriateness of the long-used, carbapenem-sparing, institutional antimicrobial protocol used for patients with FN.

### Etiology

The causes of NF can be divided into microbiologically-defined infection, clinically-defined infection and fever of unknown origin. A microbiologically-defined infection is identified in only 40-50% of patients presenting with febrile neutropenia, with only 10-30% having bacteremia, while the clinically-documented NF is diagnosed when the microbiologic workup is negative but there is a high clinical suspicion of infection based on physical examination and radiological findings (11, 12).

**Figure 1:**
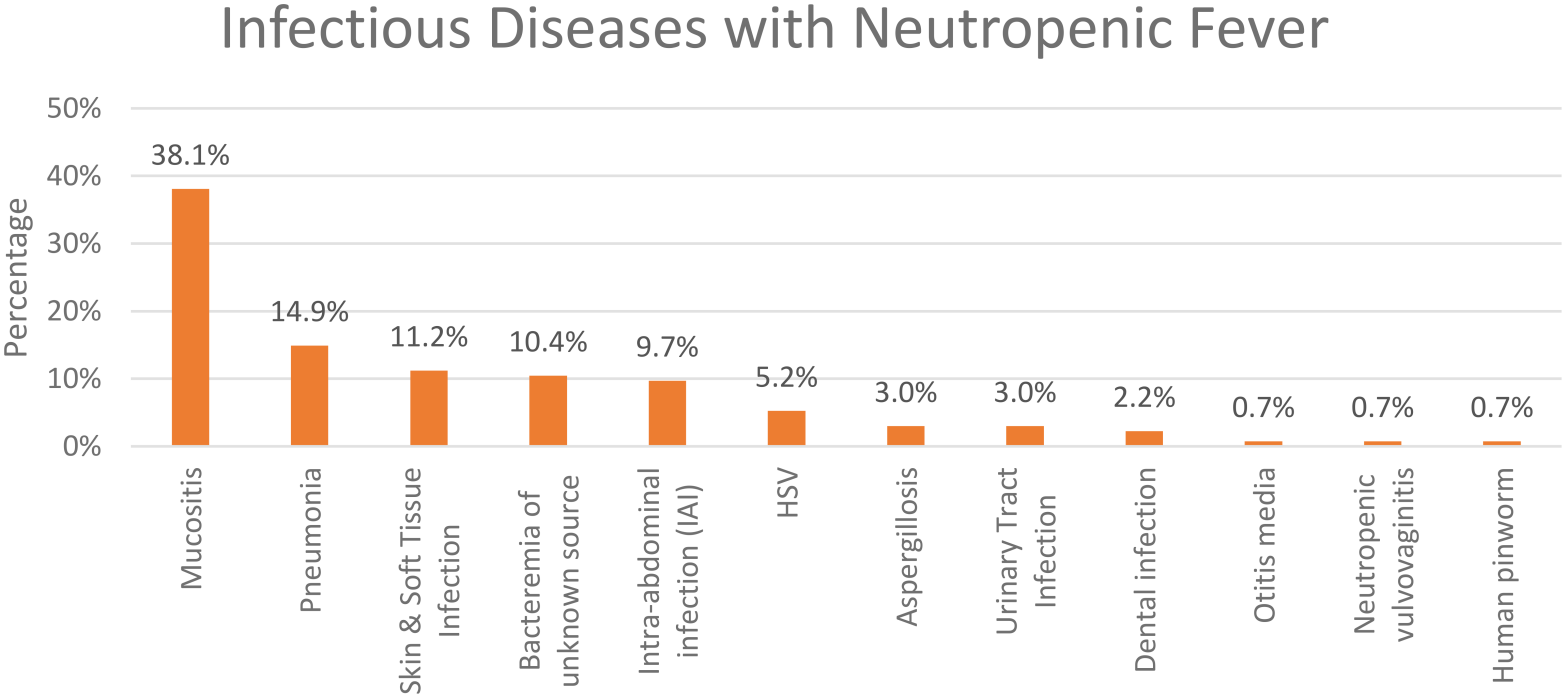
Source of infection in the studied sample.

Clinically-documented neutropenic fever is often due to incomplete medical examination, or a clerical error caused by untimely collection of cultures. A third, often common cause of fever in cancer patients might be non-infectious cause of fever with neutropenia, often grouped together with fever of unclear origin. Patients who respond well to empiric antibiotics might harbor a hidden infection. Other, alternative causes of fever with neutropenia include mucositis secondary to chemotherapy, neoplastic fever, transfusion-related fever, drug-induced fever, cytokine release storm, graft-versus-host disease or fever of unknown origin (12). In our study, 38.1% of our patients had concurrent mucositis, while the most frequently-identified source of fever was pneumonia, which accounted for 14.9% of cases. Skin and soft tissue infections accounted for 7.9% of cases, setting the percentage of non-infectious causes of NF at 91.6%, which is much higher than the worldwide average, as an Australasian study put the percentage of clinically-defined infection to be as high as 70% (13). **Figure 1** shows the underlying cause of fever in the cases of neutropenic fever.

### Microbiology

In our study, only 18 out of the 208 studied cases had positive blood cultures, for which the three studied regimens had variable appropriateness rates, however, there was no significant difference in their coverage rates. The actual antibiotic treatment given to bacteremic patients was variable in appropriateness but with no significant difference (*p* = 0.005), however, this conclusion is limited by the small number of positive blood cultures to assess for appropriateness. It is worth noting that our institutional regimen (ceftazidime with amikacin or ciprofloxacin) had a similar coverage rate as monotherapy with piperacillin-tazobactam, the later coverage rate increased after the addition of vancomycin to the regimen due to extended coverage of Gram-positive bacteria. However, this practice is not recommended as a standard part of the initial antibiotic regimen for fever and neutropenia (3). Within the limitations of this retrospective analysis, the institutional regimen demonstrated observed outcomes comparable to those reported for internationally recommended approaches; however, These results should be regarded as exploratory and hypothesis-generating rather than confirmatory.

Many centers reported a predominance of Gram-positive organisms isolated from patients with FN, however, our study showed predominance of Gram-negative bacteria. The relative predominance of Gram-negative and Gram-positive bacteremia is often dependent on the definition of blood-stream infection (BSI), patient setting, period of reporting and the use of fluoroquinolones prophylaxis (FQP), as FQP reduces the incidence of Gram-negative bacteria (13). The most frequently isolated pathogen in our study was *Enterobacterales*, followed by Gram-positive *Streptococci* species, as FQP is not a common practice at our center. Predominance of Gram-negative bacteremia was similar to other studies in our geographic region, as a study from Saudi Arabi pointed a predominance of Gram-negative pathogens at a percentage of 65.94% (14).

Regarding multidrug resistant organisms, there is no consensus regarding the empiric treatment in patients with NF who are known to be colonized or previously infected by a multidrug resistant Gram-negative organism, however, American and European guidelines suggest empiric colistin with a β-lactam or (or one of tigecycline, aminoglycoside, fosfomycin) (15). This is also practiced in our hospital, but on a case-by-case basis. In our study, seven of our patients grew ESBL Gram-negative organisms, one of them was empirically commenced on piperacillin-tazobactam, or 2^nd^ line regimen, as he was known to be colonized by ESBL. The rest were initially started on our institutional regimen then escalated according to culture results.

Invasive aspergillosis is a significant cause of morbidity and mortality in patients with FN, especially in patients with hematologic malignancies. It should be considered in patients with prolonged fever who are not responding to initial antibiotic therapy (16). In our study, 3.0% of our patients were eventually found to have invasive aspergillosis, all of whom were hematology patients.

### Escalation of therapy

Patients with FN should be evaluated on a daily basis and reassessed after 72 hours, if there are signs of clinical response, treatment should be continued for around seven days. If there is inadequate clinical improvement, treatment should be adjusted in concordance with culture and sensitivity if the pathogen is isolated. Alternatively, the patient should be screened for a localized infection and the use of granulocyte colony stimulating factor (GCS-F) should be considered. Moreover, additional bacterial cultures and serological testing should be performed, new imaging should be sought to look for occult infection. Antifungals, antivirals and anaerobic coverage should be considered. Noninfectious causes should also be sought (11). For patients with severe mucositis or neutropenic enterocolitis, it is not recommended to deescalate antibiotic therapy to target isolated organisms until bone marrow recovery due to the polymicrobial nature of these infections, which often include enteric Gram-negatives, *Enterococci* species, anaerobes and viridans group *Streptococci* (13). 74.52% of our studied patients improved on our institutional empiric therapy, inferred from resolution of neutropenia or clinical improvement of symptoms, 16.83% required escalation to piperacillin-tazobactam, with or without vancomycin. A notable strength of our protocol is that vancomycin is reserved for patients with specific clinical indications (e.g., suspected catheter-related infection, septic shock or severe Gram-positive infection) rather than administered routinely, thereby supporting antimicrobial stewardship. Only 7.21% required escalation to meropenem, with or without vancomycin. ASCO recommends assessing patients initially for a possible source of infection and daily for development of new symptoms or for non-improvement, with the antibiotic regimen tailored to their clinical status. For example, patients with abdominal symptoms may require the addition of anaerobic coverage, such as piperacillin-tazobactam or metronidazole. Antipseudomonal carbapenems, despite their excellent anaerobic coverage, are best reserved for complicated infections or drug-resistant organisms. On the other hand, carbapenems might be used initially for patients presenting with sepsis or for those who are stable initially but become septic during their stay, requiring escalation of therapy (11).

### Hematology vs. oncology

**Figure 2:**
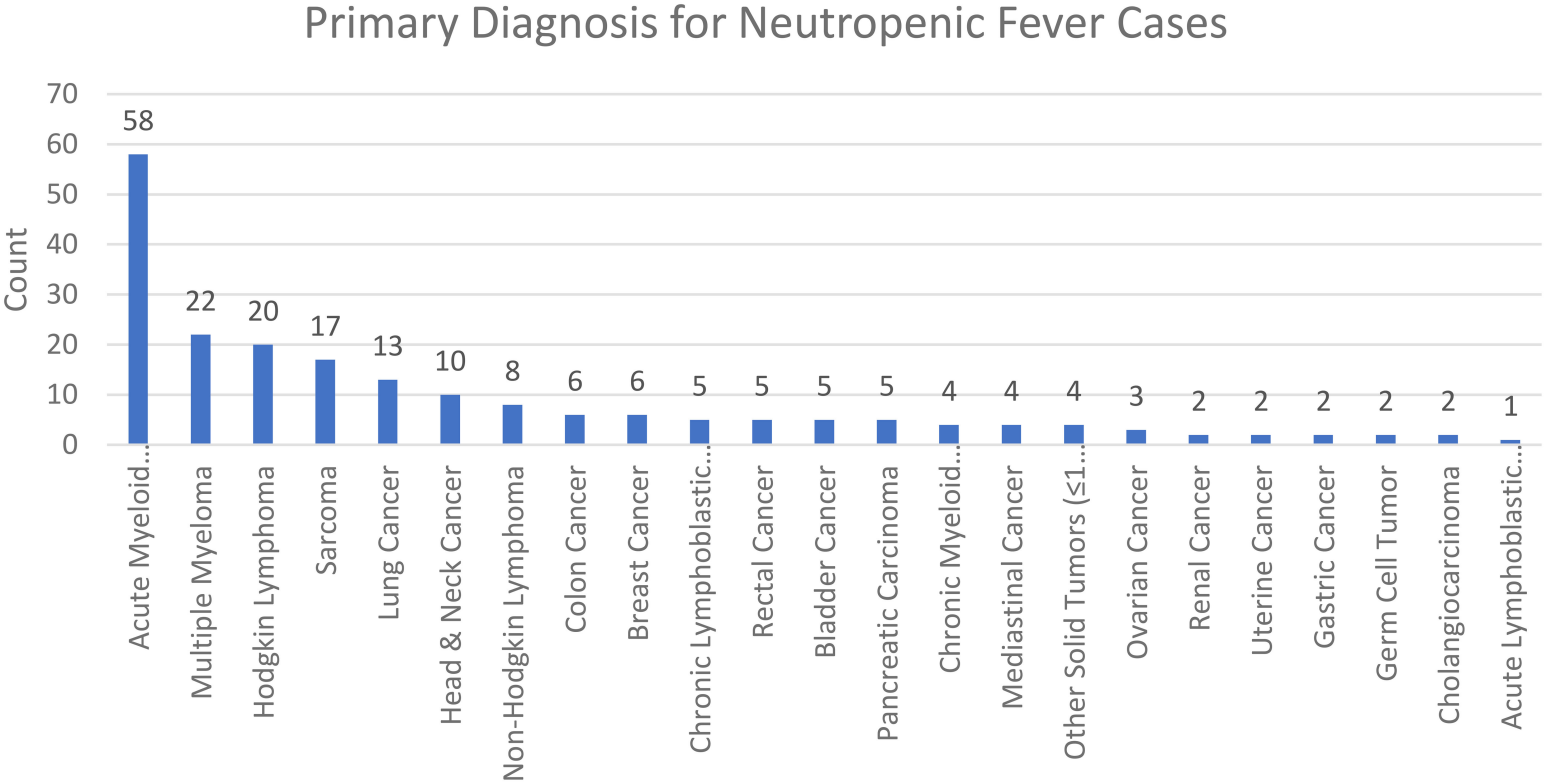
Distribution of the primary cancer in patients admitted with febrile neutropenia.

In our study, 56.7% of cases had hematological malignancies. They had a higher incidence of positive cultures (61% of positive blood cultures), 81.8% of which due to Gram-negative bacteremia. Our findings were comparable to a study from Turkey, which was comprised of 63% hematology patients compared to 37% oncology patients (17). **Figure 2** shows the distribution of primaries cancers in patients admitted with neutropenic fever.

### Clinical outcome

**Figure 3:**
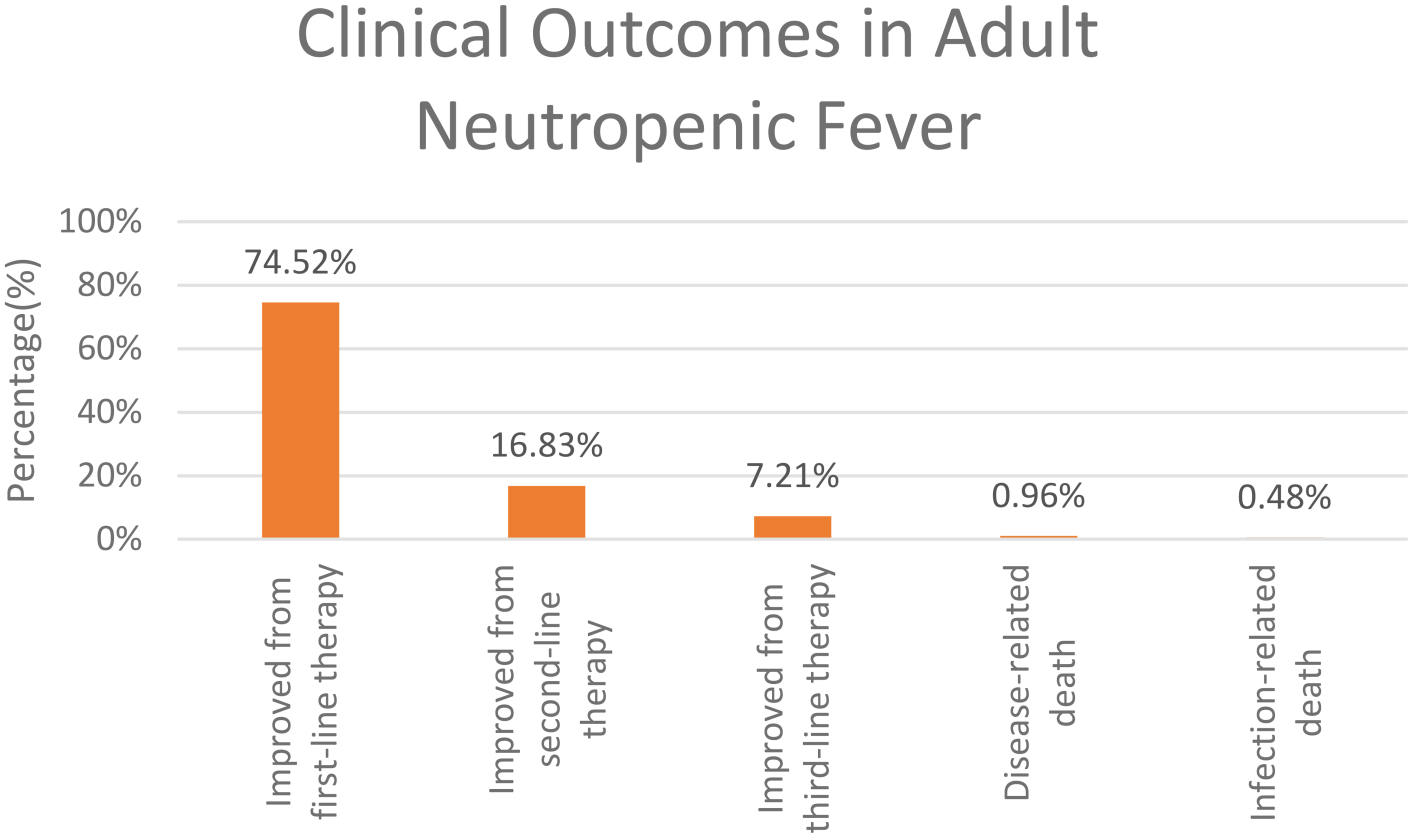
Clinical outcome of patients with febrile neutropenia.

A positive clinical outcome was defined as either resolution of symptoms after sufficient duration of antibiotics or resolution of neutropenia. Negative clinical outcome was defined as worsening of symptoms, requiring antibiotic upgrade. Death was divided into disease related and infection related. Clinical improvement was achieved in 74.5% of patients treated with 1^st^-line regimen, while 66.0% of those requiring escalation to 2^nd^ line regimen, and 83.3% of those escalated to the broadest, 3^rd^ line regimen. Given the sequential nature of treatment escalation, formal comparison between groups was not performed. **Figure 3** shows the clinical outcome of adults with neutropenic fever.

### Mortality

FN is considered an oncological emergency, with a high rate of mortality and morbidity in untreated patients. Patients with Gram-negative sepsis unopposed with proper antibiotic coverage can have a mortality rate of up to 70%, while the overall mortality rate of patients treated with empiric antibiotics can be between 4% to 20%. Higher mortality rates are seen in patients with multiple comorbidities, documented Gram-negative rod bacteremia, and tissue-invasive infections (18). In our cohort, 11 of 208 episodes (5.3%) initially managed as neutropenic fever were ultimately attributed to non-infectious etiologies, including neoplastic, rheumatologic, drug-induced and central causes. Additionally, five out of 208 episodes of neutropenic fever (2.4%) progressed to septic shock. Furthermore, 0.48% of our patients had infection-related death, while 0.96% of patients admitted with NF experienced death due to disease related complications rather than infection. Resulting in an all-cause mortality rate was 1.44%. These findings suggest that severe infectious outcomes were uncommon in our cohor; however, interpretation should remain cautious given the retrospective design and limited number of severe events.

### Limitations

Several limitations should be considered when interpreting the microbiological appropriateness analysis. First, only 18 culture-positive cases were available for evaluation, limiting statistical power and reducing the ability to draw robust comparative conclusions regarding theoretical pathogen coverage across regimens. In particular, the small number of patients requiring escalation to the broadest-spectrum regimen may have resulted in imprecise estimates and increased susceptibility to type II error. Second, regimen allocation was not randomized but reflected a predefined sequential escalation protocol based on clinical response and disease severity, introducing inherent selection bias and preventing direct comparison of regimen effectiveness. Consequently, the absence of statistically significant differences should not be interpreted as evidence of equivalent performance between regimens because repeated admissions from the same patient were analyzed as independent episodes, some observations may not have been completely independent. Although repeat admissions represented a minority of episodes, this could have introduced a small degree of correlation that was not accounted for statistically. Finally, microbiological appropriateness was assessed through simulated theoretical coverage of isolated pathogens and should not be considered a direct measure of clinical effectiveness. Confidence intervals should therefore be interpreted alongside point estimates to reflect the uncertainty surrounding these findings.

## Conclusion

In this retrospective cohort of 208 episodes of febrile neutropenia, the institutional stepwise empiric antibiotic protocol was associated with favorable clinical outcomes, with the majority of patients responding to first-line therapy and only a minority requiring sequential escalation to broader-spectrum regimens. These findings support the effectiveness of a carbapenem-sparing, stewardship-oriented approach in our institution.

As a secondary analysis, simulated microbiological assessment of the 18 culture-positive episodes demonstrated progressively higher theoretical pathogen coverage with broader-spectrum regimens. However, these findings should be interpreted with considerable caution because of the small number of culture-positive cases, the highly imbalanced distribution of administered regimens, and the sequential, non-independent treatment allocation. Accordingly, these microbiological results should be regarded as exploratory and hypothesis-generating rather than evidence of comparative clinical superiority.

Overall, our findings support the continued use of a stepwise escalation strategy that balances favorable clinical outcomes with antimicrobial stewardship principles while minimizing unnecessary exposure to broad-spectrum antibiotics. Larger prospective, multicenter studies are needed to validate the microbiological findings and determine whether broader theoretical pathogen coverage translates into improved clinical outcomes.

## Data Availability

The raw data supporting the conclusions of this article will be made available by the authors, without undue reservation.
